# Examining non-medical prescribing trends in New Zealand: 2016–2020

**DOI:** 10.1186/s12913-021-06435-y

**Published:** 2021-05-03

**Authors:** Rakhee Raghunandan, Carlo A. Marra, June Tordoff, Alesha Smith

**Affiliations:** School of Pharmacy, University of Otago, PO Box 56, Dunedin, Otago 9054 New Zealand

**Keywords:** Non-medical prescribing, Prescribing practice, Workforce, Healthcare, New Zealand

## Abstract

**Background:**

Population growth and general practitioner workforce constraints are creating increasing demand for health services in New Zealand (NZ) and internationally. Non-medical prescribing (NMP) is one strategy that has been introduced to help manage this. Little is known about the NMP practice trends in NZ. The aim of this study was to provide a current overview of the scale, scope, and trends of NMP practice in NZ.

**Methods:**

All claims for community dispensed medicines prescribed by a non-medical prescriber were extracted from the NZ Pharmaceutical Collection for the period 2016–2020. Patient demographics were retrieved from the Primary Health Organisation enrolment collection. These national databases contain prescription information for all subsidised community pharmacy medicines dispensed and healthcare enrolment data for 96% of New Zealanders.

**Results:**

The proportion of prescriptions written by all NMP providers and patients receiving NMP prescriptions increased each year from 1.8% (2016) to 3.6% (2019) and 8.4% (2016) to 14.4% (2019) respectively. From 2016 to 2019, the proportion of NMP patients who had at least one NMP prescription increased from 26% to 39% for nurse prescribers, from 1% to 9% for pharmacist prescribers, from 2% to 3% for dietitian prescribers, and decreased from 47% to 22% for dentists, and from 20% to 12% for midwives. The most commonly prescribed medicines were antibiotics (amoxicillin, amoxicillin with clavulanic acid, and metronidazole), and analgesics (paracetamol, and codeine phosphate). While some NMP providers were prescribing for patients with greater health needs, all NMP providers could be better utilised to reach more of these patients.

**Conclusions:**

This study highlights that although the NMP service has been implemented in NZ, it has yet to become mainstream healthcare practice. This work provides a baseline to evaluate the NMP service moving forward and enable policy development. Improved implementation and integration of primary care NMP services can ensure continued access to prescribing services and medicines for our communities.

## Background

As in other countries, non-medical prescribing (NMP) was introduced in New Zealand (NZ) to address the increased demand for healthcare services and diminishing access to prescribing services and medicines [1–3]. NMP is the legislative extension of prescribing rights to health professionals other than medical doctors, thereby enabling them to prescribe medicines classified as prescription medicines, and facilitate timely access to medicines [4]. This is particularly significant as 49% of general practitioners (GPs) in NZ work part time and 47% of GPs intend to retire from general practice in the next 10 years [5]. The inequity of access to medicines in primary care in NZ is well documented and includes issues such as physical and timely access to a prescriber/prescription, and the direct and indirect costs associated with consulting a prescriber [6].

NMP is still a relatively new practice in NZ and is changing significantly with the number of non-medical health professional groups gaining prescribing rights increasing. NZ non-medical prescribers include dentists, midwives, nurse prescribers (nurse practitioners and registered nurse prescribers), pharmacist prescribers, optometrists and dietitian prescribers [7]. In NZ dentists, nurse practitioners, midwives, and optometrists prescribe independently without a medical prescriber, while pharmacist prescribers, registered nurse prescribers, and dietitian prescribers all prescribe collaboratively with the involvement of a medical prescriber [7]. There is some NZ research examining the rates of NMP in NZ [8], and the prescribing practice of nurse practitioners [9–11] and dentists [12]. There is paucity of research that encompasses all NMP and current research is very profession-specific. Little is known about the current utilisation of NMP services and the prescribing practice of all non-medical prescribers in NZ. Eliciting this type of information will help describe the current impact/contribution of NMP in primary care NZ, and can contribute to workforce development to enable efficient and sustainable NMP services in NZ.

This study aims to provide a current overview of non-medical prescribers’ prescribing trends in NZ with specific objectives to determine the:

- contribution and trends of all non-medical prescribing to overall prescribing from 2016 to 2020;
- contribution and trends of each NMP health professional group to all non-medical prescribing from 2016 to 2020;
- demographics of patients who receive prescriptions from non-medical prescribers;
- most frequently prescribed therapeutic groups and medicines by non-medical prescribers.

## Methods

### Study design

This retrospective nationwide observational study used the NZ Ministry of Health Pharmaceutical Collection and the Primary Health Organisation (PHO) Enrolment databases [13, 14]. These databases capture 96% of all New Zealanders, as approximately 4% of the NZ population are not enrolled at a primary care practice [15] and are not captured in these two databases.

Medicines and therapeutic products that are publicly funded in NZ are listed in the NZ Pharmaceutical Schedule (community and hospital schedules), which is managed by the Pharmaceutical Management Agency, PHARMAC [16]. PHARMAC is an independent statutory organisation responsible directly to the NZ Minister of Health and works with the Ministry of Health [17]. PHARMAC determines which medicines and therapeutic products will be fully and/or partly subsidised by public funding in NZ [18]. The Pharmaceutical Collection, which is jointly owned by the Ministry of Health and PHARMAC, is a data warehouse that records the claims submitted by all pharmacies in NZ for the reimbursement of the subsidised medicines that have been dispensed to all patients [19]. The PHO Enrolment Collection is a national database that holds primary care patient enrolment data [20].

The Pharmaceutical Collection was searched between 1 January 2016 to 30 June 2020 (4.5 years) using the distinct provider type codes to identify dispensed medicines prescribed by: dentists, nurse prescribers (which includes nurse practitioners and registered nurse prescribers), optometrists, midwives, pharmacist prescribers, and dietitian prescribers. The number of distinct enrolled patients who were dispensed a medicine from a NMP and the number of original prescriptions dispensed were also determined. The distinct original prescription dispensing refers to each single item prescribed by a prescriber on a prescription form to a patient, and does not include any repeat dispensing that occurred from a prescription. The distinct patients were identified using the encrypted National Health Index (NHI) number, which is unique for each patient and remains the same in each health-related database across a patient’s lifetime and allows the linking of collections. The following patient sociodemographic details were also retrieved from the PHO Enrolment Collection: age, gender, ethnicity, NZ Deprivation Index (NZDep2013 score), and District Health Board (DHB) provider. Data for the 100 most dispensed medicines by therapeutic group and medicine chemical name (as listed in the community Pharmaceutical Schedule) [16] for overall NMP and each NMP provider were also retrieved from the Pharmaceutical Collection.

### Analysis

Descriptive statistical analyses, using Excel 2016®, were conducted on the aggregated data set, to determine NMP contribution to overall community dispensed medicines in NZ, NMP by non-medical prescriber provider type (e.g. nurse prescriber, dentist, etc.), the demographics of the patients who received prescriptions from non-medical prescribers, and summarise the types of medicines prescribed by non-medical prescribers in NZ.

The study analysed patients’ DHB, deprivation and self-reported ethnicity data. The following Statistics New Zealand reporting standards for ethnicity were used: Asian, Māori, MELAA (defined as Middle Eastern, Latin American, and African), NZ European/European, Other, and Pacific Peoples. The NZDep2013 score combines census data relating to income, home ownership, employment, qualifications, family structure, housing, access to transport and communications [21]. The NZDep2013 index groups deprivation scores into deciles, where 1 represents the areas with the least deprived scores and 10 the areas with the most deprived scores [21]. DHB rural populations were identified from a Ministry of Health commissioned report [22]. DHBs were categorised as ‘rural’ if they had a greater than 20% rural population.

## Results

This study was approved by the University of Otago Human Ethics Committee (reference HD20/029).

### NMP and non-medical prescriber providers

The number of dispensed prescriptions written by all healthcare prescribers (medical and non-medical) increased by 10.8% from 2016 to 2019 (78,056,369 prescriptions vs 86,514,345). The number of patients who had a prescription issued by all healthcare prescribers (medical and non-medical) increased by 4.7% from 2016 to 2019 (3,356,850 vs 3,514,106) (Table 1). The number of medical prescriptions dispensed also increased from 2016 to 2019 (76,476,931 vs 83,227,282).

**Table 1:** Summary of NZ NMP by number of original prescriptions dispensed and patients from 2016 to 2020

Calendar year	Medical prescriptions dispensed	NMP prescriptions dispensed	All (medical and NMP) prescriptions dispensed^**a**^	NMP prescriptions as % of all prescriptions	Medical patients dispensed to	NMP patients dispensed to	All (medical and NMP) patients dispensed to^**a**^	NMP patients as % of all patients dispensed to
2016	76,476,931	1,407,028	78,056,369	1.8%	3,318,498	282,841	3,356,850	8.4%
2017	81,006,690	1,851,714	83,023,775	2.2%	3,432,519	347,441	3,477,274	10.0%
2018	84,263,278	2,610,400	87,075,543	3.0%	3,482,168	445,528	3,538,311	12.6%
2019	83,227,282	3,101,161	86,514,345	3.6%	3,450,211	506,945	3,514,106	14.4%
2020 half year (1st Jan–30th June)	33,163,443	1,304,021	34,535,777	3.8%	2,574,434	239,478	2,641,302	9.1%
**2016–2020**	**358,137,624**	**10,274,324**	**369,205,809**	**2.8%**	**16,257,830**	**1,822,233**	**16,527,843**	**11.0%**

^**a**^ = totals may not sum the Medical and NMP columns due to patients getting a prescription from more than one prescriber (i.e. medical and NMP providers) and/or data classification error in databases

The annual proportion of NMP prescriptions for all prescriptions dispensed and NMP patients for all patients dispensed to, has increased over the past 4 years (i.e. 1.8% in 2016 to 3.6% in 2019 for NMP prescriptions and 8.4% in 2016 to 14.4% in 2019 for NMP patients respectively) (Table 1), but not at the same rates for all prescribing (i.e. increases of 10.8% for prescriptions and 4.7% for patients).

**Fig. 1:**
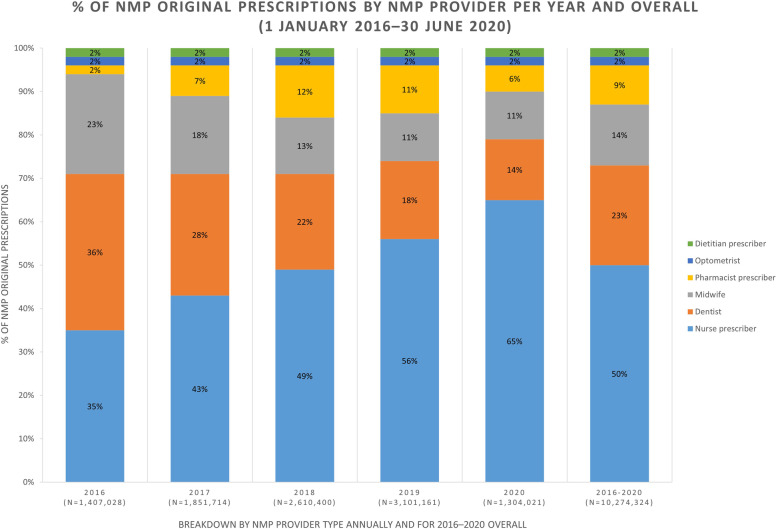
Contribution by each NMP provider type towards NMP prescriptions each year and overall from 1 January 2016–30 June 2020

Nurse prescribers were the largest NMP contributors (1.40%) to all prescriptions (medical and non-medical) dispensed between 2016 and 2020, followed by dentists (0.64%), midwives (0.39%), and pharmacist prescribers (0.25%). Optometrists and dietitian prescribers were the smallest contributors equally (0.06%). Nurse prescribers increased their proportion of all NMP prescriptions from 35% (2016) to 56% (2019), and they contributed 50% of all NMP prescriptions for the four-and-a-half-year period (2016–2020) (Fig. 1). Dentists had the largest proportion of NMP prescriptions in 2016 (36%) and the second largest (although decreasing) in 2019 (18%), and by mid-2020 this was 14%. Pharmacist prescribers’ contributions increased from 2% in 2016 to 11% in 2019, and contributed 9% of all NMP prescriptions for the four-and-a-half-year period (2016–2020).

### Patients

**Fig. 2:**
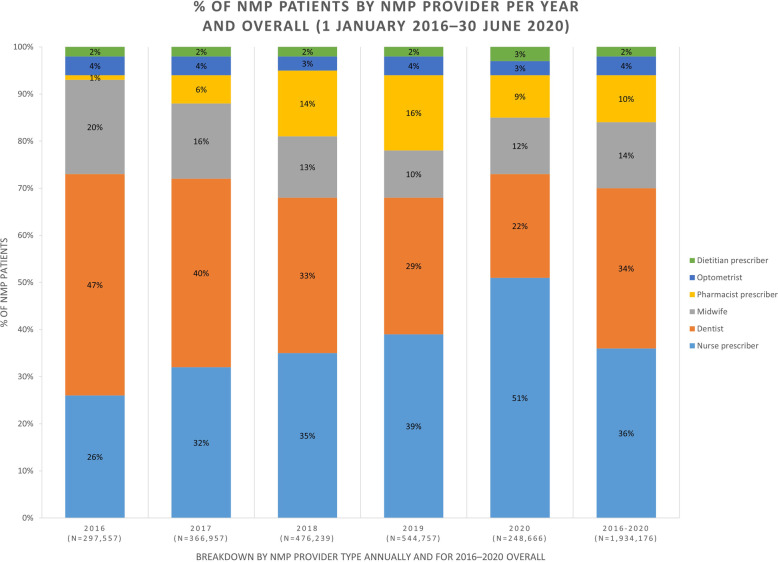
Contribution by each NMP provider type towards NMP patients each year and overall from 1 January 2016–30 June 2020

Nurse and pharmacist prescribers were prescribing for an increasing proportion of NMP patients from 2016 to 2019 (Fig. 2). Nurse prescribers prescribed for 36% of all NMP patients and pharmacist prescribers prescribed for 10% of all NMP patients for the four-and-a-half-year period (2016–2020). From 2016 to 2020, 47–22% of NMP patients had prescriptions from dentists; 20–12% from midwives; 4–3% from opticians; and 2–3% from dietitians.

Table 2 presents the sociodemographic characteristics of the patients who had a NMP prescription dispensed between 1 January 2016–30 June 2020, and summarises these findings in terms of each specific non-medical provider type and NMP providers overall. Individual patients may have received a prescription from multiple NMP and medical providers (e.g. dentist, midwife, and GP). Hence, the number of original prescriptions and/or number of patients differs for many categories in the analyses.

**Table 2:** Summary of sociodemographic characteristics of patients seen by all NMP providers and each specific NMP provider type for period 1 January 2016–30 June 2020

Variable:	Dentist	Nurse prescriber	Optometrist	Midwife	Pharmacist prescriber	Dietitian prescriber	All NMP providers
**1). Gender:**	***N*** **= 474,312 (%)**	***N*** **= 387,816 (%)**	***N*** **= 46,615 (%)**	***N*** **= 172,483 (%)**	***N*** **= 133,093 (%)**	***N*** **= 31,880 (%)**	***N*** **= 1,089,293 (%)**
Female	250,652 (52.8%)	241,769 (62.3%)	27,394 (58.8%)	160,157 (92.9%)	89,578 (67.3%)	15,662 (49.1%)	663,908 (60.9%)
Male	223,660 (47.2%)	146,047 (37.7%)	19,221 (41.2%)	12,326 (7.1%)	43,515 (32.7%)	16,218 (50.9%)	425,385 (39.1%)
**2). Age (years):**	***N*** **= 554,007**	***N*** **= 520,488**	**N = 55,041**	***N*** **= 227,500**	***N*** **= 169,683**	***N*** **= 42,764**	**N = 1,483,156**
Mean (SD)	46.6 (20)	38.9 (25)	53.5 (22)	26.7 (10.4)	52.9 (23.3)	50.8 (30.3)	42.4 (23.1)
Median (IQR)	48 (30–62)	36 (19–40)	57 (38–71)	29 (24–33)	59 (31–72)	60 (20–76)	39 (25–61)
Range	0–101	0–101	0–101	0–55	0–101	0–101	0–101
**3). NZDep2013 score:**	***N*** **= 447,345**	***N*** **= 363,634**	**N = 44,647**	***N*** **= 161,243**	***N*** **= 126,702**	***N*** **= 23,754**	***N*** **= 1,017,132**
Mean (SD)	5.5 (2.9)	6.4 (2.8)	5 (2.8)	5.9 (2.9)	5.7 (2.9)	5.8 (2.9)	5.8 (2.9)
Median (IQR)	6 (3–8)	7 (4–9)	5 (2–7)	6 (3–9)	6 (3–8)	6 (3–8)	6 (3–8)
Range	0–10	0–10	0–10	0–10	0–10	0–10	0–10
**4). NZDep2013 decile:**	**N = 474,338 (%)**	**N = 387,834 (%)**	**N = 46,616 (%)**	**N = 172,493 (%)**	**N = 133,102 (%)**	**N = 31,881 (%)**	**N = 1,089,353 (%)**
Decile 1–2	89,128 (18.8%)	47,659 (12.3%)	11,276 (24.2%)	26,638 (15.5%)	23,562 (17.7%)	4086 (12.8%)	178,507 (16.4%)
Decile 3–4	84,601 (17.8%)	56,973 (14.7%)	9102 (19.5%)	28,253 (16.4%)	22,604 (17.0%)	4136 (13.0%)	180,375 (16.6%)
Decile 5–6	88,545 (18.7%)	64,723 (16.7%)	9282 (19.9%)	29,899 (17.3%)	23,582 (17.7%)	4438 (13.9%)	191,857 (17.6%)
Decile 7–8	93,044 (19.6%)	81,669 (21.1%)	8328 (17.9%)	35,161 (20.4%)	28,880 (21.7%)	5615 (17.6%)	218,952 (20.1%)
Decile 9–10	92,027 (19.4%)	112,610 (29.0%)	6659 (14.3%)	41,292 (23.9%)	28,074 (21.1%)	5479 (17.2%)	247,441 (22.7%)
Null	26,993 (5.7%)	24,200 (6.2%)	1969 (4.2%)	11,250 (6.5%)	6400 (4.8%)	8127 (25.5%)	72,221 (6.6%)
**5). Ethnicity:**	***N*** **= 471,313 (%)**	**N = 387,203 (%)**	**N = 46,330 (%)**	**N = 172,419 (%)**	***N*** **= 132,629 (%)**	**N = 31,757 (%)**	**N = 1,085,533 (%)**
European	335,774 (71.3%)	244,723 (63.2%)	36,407 (78.6%)	92,836 (53.8%)	98,434 (74.2%)	23, 659 (74.5%)	727,777 (67.0%)
Māori	60,477 (12.8%)	83,756 (21.6%)	2676 (5.8%)	30,434 (17.6%)	15,872 (12.0%)	4171 (13.1%)	171,882 (15.8%)
Asian/Indian	44,790 (9.5%)	32,534 (8.4%)	5218 (11.2%)	33,598 (19.5%)	11,446 (8.6%)	2466 (7.8%)	113,582 (10.5%)
Pacific peoples	22,309 (4.7%)	20,299 (5.3%)	1284 (2.8%)	11,309 (6.6%)	4645 (3.5%)	1169 (3.7%)	54,011 (5.0%)
Other (including Middle Eastern/Latin American/African)	7963 (1.7%)	5891 (1.5%)	745 (1.6%)	4242 (2.5%)	2232 (1.7%)	292 (0.9%)	18,281 (1.7%)
**6). NZ District Health Board (DHB) domicile:**	***N*** **= 484,698 (%)**	***N*** **= 394,573 (%)**	**N = 47,310 (%)**	***N*** **= 183,230 (%)**	***N*** **= 136,771 (%)**	***N*** **= 32,994 (%)**	**N = 1,137,031 (%)**
Northland^a^	17,548 (3.6%)	23,841 (6.0%)	878 (1.9%)	6315 (3.4%)	3561 (2.6%)	1321 (4.0%)	48,445 (4.3%)
Waitematā	56,931 (11.8%)	25,168 (6.4%)	6975 (14.7%)	24,521 (13.4%)	13,969 (10.2%)	2881 (8.8%)	116,849 (10.3%)
Auckland	49,557 (10.2%)	20,340 (5.2%)	7931 (16.8%)	18,335 (10.0%)	13,387 (9.8%)	3214 (9.8%)	103,098 (9.1%)
Counties Manukau	49,799 (10.3%)	41,844 (10.6%)	4151 (8.8%)	22,885 (12.5%)	9729 (7.1%)	2357 (7.1%)	117,157 (10.3%)
Waikato^a^	51,463 (10.6%)	27,700 (7.0%)	4811 (10.2%)	17,232 (9.4%)	24,310 (17.8%)	3788 (11.5%)	113,218 (10.0%)
Bay of Plenty	25,073 (5.2%)	27,668 (7.0%)	3707 (7.8%)	8229 (4.5%)	5417 (4.0%)	1049 (3.2%)	62,806 (5.5%)
Taranaki^a^	12,489 (2.6%)	9312 (2.4%)	827 (1.8%)	3974 (2.2%)	1531 (1.1%)	1419 (4.3%)	26,194 (2.3%)
Lakes	11,257 (2.3%)	24,681 (6.3%)	825 (1.7%)	4349 (2.4%)	3829 (2.8%)	462 (1.4%)	39,710 (3.5%)
Tairāwhiti^a^	5420 (1.1%)	3116 (0.8%)	191 (0.4%)	1946 (1.1%)	1054 (0.8%)	410 (1.2%)	10,865 (1.0%)
Whanganui	5730 (1.2%)	4610 (1.2%)	583 (1.2%)	2544 (1.4%)	1545 (1.1%)	434 (1.3%)	13,954 (1.2%)
Midcentral	18,796 (3.9%)	41,080 (10.4%)	1434 (3.0%)	7643 (4.2%)	3834 (2.8%)	1532 (4.6%)	64,252 (5.6%)
Hawke’s Bay	14,648 (3.0%)	23,177 (5.9%)	1298 (2.7%)	6735 (3.7%)	8906 (6.5%)	1165 (3.5%)	48,479 (4.3%)
Capital and Coast	33,191 (6.8%)	13,944 (3.5%)	4110 (8.7%)	9873 (5.4%)	11,016 (8.0%)	1968 (6.0%)	66,033 (5.8%)
Hutt Valley	16,115 (3.3%)	5964 (1.5%)	1677 (3.6%)	5541 (3.0%)	4185 (3.1%)	885 (2.7%)	30,800 (2.7%
Wairarapa^a^	4676 (1.0%)	10,745 (2.7%)	234 (0.5%)	1754 (0.9%)	2053 (1.5%)	379 (1.2%)	17,246 (1.5%)
Nelson & Marlborough^a^	15,183 (3.1%)	14,673 (3.7%)	714 (1.5%)	4838 (2.6%)	4296 (3.1%)	837 (2.5%)	35,666 (3.1%)
West Coast^a^	4171 (0.9%)	4093 (1.0%)	302 (0.6%)	1363 (0.7%)	304 (0.2%)	309 (0.9%)	9227 (0.8%)
Canterbury	54,350 (11.2%)	29,910 (7.6%)	3021 (6.4%)	21,202 (11.6%)	15,064 (11.0%)	5391 (16.3%)	115,353 (10.1%)
South Canterbury^a^	3939 (0.8%)	6123 (1.5%)	191 (0.4%)	2231 (1.2%)	617 (0.5%)	594 (1.8%)	12,500 (1.1%)
Southern^a^	34,362 (7.1%)	36,584 (9.3%)	3450 (7.3%)	11,720 (6.4%)	8164 (6.0%)	2599 (7.9%)	85,179 (7.5%)

^a^ = rural DHBs (with a greater than 20% rural population) [22]

*IQR* Inter-quartile range; *SD* standard deviation

While the majority of patients seen by NMP providers were female (60.9%), dentists and dietitian prescribers prescribed for almost equal proportions of female and male patients (Table 2). During the period 2016–2020, the median age for the NMP patients was 39 years (IQR 25–61 years). The youngest patients were seen by midwives (median = 29, IQR 24–33), followed by nurse prescribers (median = 36, IQR 19–40), and dentists (median = 48, IQR 30–62). The patients’ median age was 57–60 years for optometrists (median = 57, IQR 38–71), pharmacist prescribers (median = 59, IQR 31–72), and dietitian prescribers (median = 60, IQR 20–76).

The NZDep2013 score was recorded for 1,017,132 NMP patients with a median score of 6 (IQR 3–8). In total, 466,393 NMP patients (42.8%) were living in the more deprived areas (i.e. NZDep2013 decile 7–8 and above). During 2016–2020, optometrists saw patients living in the less deprived areas (NZDep2013 median score = 5, IQR 2–7) and nurse prescribers saw patients living in more deprived areas (NZDep2013 median score = 7, IQR 4–9).

For all NMP providers overall, 171,882 (15.8%) patients self-identified as Māori and 54,011 (5.0%) as Pacific peoples. Nurse prescribers prescribed for the highest proportion of Māori patients (83,756 (21.6%)) and optometrists for the lowest proportion of Māori patients (2676 (5.8%)). Midwives prescribed for the highest proportion of Pacific patients (11,309 (6.6%)) and optometrist prescribers prescribed for the lowest proportion of Pacific patients (1284 (2.8%)). Twelve percent of pharmacist prescribers’ patients self-identified as Māori and 3.5% as Pacific.

The five DHBs with the most patients receiving a prescription from a NMP provider were: Counties Manukau (117,157 patients (10.3%)), Waitematā (116,849 (10.3%)), Canterbury (115,353 (10.1%)), Waikato DHB (113,218 (10.0%)), and Auckland (103,098 (9.1%)). Most rural DHBs had lower numbers of NMP patients (e.g. Wairarapa 17,246 (1.5%) and South Canterbury 12,500 (1.1%)), and included the two DHBs with the least NMP patients which were West Coast (9227 (0.8%)) and Tairāwhiti (10,865 (1.0%)).

### Prescriptions

Based on the 100 most frequently prescribed therapeutic groups by NMP providers, we ascertained the top five therapeutic products that were most prescribed by NMP providers during the period from 1 January 2016–30 June 2020. For all NMP providers, the two most frequently prescribed therapeutic groups were antibacterials with 1,802,812 dispensings (23.4%), followed by analgesics (1,096,706 (14.3%)). Antibacterials were also the most prescribed therapeutic group by dentists (1,263,218 (53.8%)), and the second most prescribed therapeutic group by nurse prescribers (432,657 (8.4%)) and optometrists (5320 (2.5%)). Analgesics were the most prescribed therapeutic group by nurse prescribers (534,387 (10.4%)), and the second most prescribed therapeutic group by dentists (418,768 (17.8%)) and midwives (115,852 (7.7%)). The analgesic therapeutic group included medicines such as paracetamol, codeine phosphate, and tramadol, but excluded ibuprofen and diclofenac, which are categorised as non-steroidal anti-inflammatory agents in the Pharmaceutical Collection data.

The top five most prescribed therapeutic groups by pharmacist prescribers were contraceptives-hormonal with 159,013 dispensings (17.9%), followed by vaccinations (128,332 (14.4%)), treatments for substance dependence (113,378 (12.7%)), lipid-modifying agents (38,199, (4.3%)), and agents affecting the renin-angiotensin system (37,353 (4.2%)). Unsurprisingly, the eye preparation therapeutic group was most prescribed by optometrists (95.6%), and all the top five therapeutic groups prescribed by dietitian prescribers related to nutrition.

Based on the 100 most frequently prescribed medicines by NMP providers, Table 3 presents the top ten medicines prescribed during the study period. For all NMP providers, the most frequently prescribed medicine was amoxicillin (902,786 dispensings, 17.5%), followed by paracetamol (617,500 (12.0%)), and amoxicillin with clavulanic acid (458,519 (8.9%)). Amoxicillin was most prescribed medicine by dentists (737,840 dispensings, 32.6%), and the third most prescribed medicine by nurse prescribers (140,754 (6.2%)). Paracetamol was the most prescribed medicine by nurse prescribers (362,511 (15.8%)), and featured in the top ten medicines prescribed by dentists, midwives and pharmacist prescribers. Compared with the top ten medicines prescribed by dentists and nurses, pharmacist prescribers were prescribing more medicines associated with long term conditions (i.e. atorvastatin, metoprolol succinate, omeprazole, aspirin, salbutamol, and metformin).

**Table 3:** Top ten medicines (out of the 100 most frequently prescribed medicines) by each specific NMP provider type and all NMP providers for period 1 January 2016–30 June 2020

Rank	Dentist *N* = 2,265,531 dispensings (%)	Nurse prescriber *N* = 2,288,317 dispensings (%)	Optometrist *N* = 202,067 dispensings (%)	Midwife *N* = 1,280,388 dispensings (%)	Pharmacist prescriber *N* = 648,746 dispensings (%)	Dietitian prescriber *N* = 170,703 dispensings (%)	All NMP providers *N* = 5,148,109 dispensings (%)
**1**	Amoxicillin 737,840 (32.6%)	Paracetamol 362,511 (15.8%)	Olopatadine 44,720 (22.1%)	Potassium iodate 308,416 (24.1%)	Levonorgestrel 154,036 (23.7%)	Oral feed (powder) 48,938 (28.7%)	Amoxicillin 902,786 (17.5%)
**2**	Amoxicillin with clavulanic acid 384,657 (17.0%)	Ibuprofen 140,901 (6.2%)	Chloramphenicol 25,002 (12.4%)	Ferrous fumarate 174,317 (13.6%)	Influenza vaccine 128,332 (19.8%)	Oral feed 1.5 kcal/ml 31,333 (18.4%)	Paracetamol 617,500 (12.0%)
**3**	Metronidazole 211,390 (9.3%)	Amoxicillin 140,754 (6.2%)	Fluorometholone 19,789 (9.8%)	Paracetamol 101,159 (7.9%)	Nicotine 112,871 (17.4%)	Paediatric oral feed 1 kcal/ml 12,031 (7.0%)	Amoxicillin with clavulanic acid 458,519 (8.9%)
**4**	Ibuprofen 187,223 (8.3%)	Atorvastatin 135,937 (5.9%)	Prednisolone acetate 15,697 (7.8%)	Folic acid 59,875 (4.7%)	Atorvastatin 25,124 (3.9%)	Oral feed with fibre 1.5 kcal/ml 7063 (4.1%)	Ibuprofen 386,182 (7.5%)
**5**	Codeine phosphate 139,664 (6.2%)	Omeprazole 129,286 (5.6%)	Latanoprost 12,374 (6.1%)	Ferrous sulphate 59,420 (4.6%)	Paracetamol 20,166 (3.1%)	Fat supplement 6212 (3.6%)	Potassium iodate 377,905 (7.3%)
**6**	Paracetamol 133,031 (5.9%)	Salbutamol 122,762 (5.4%)	Sodium Fusidate [fusidic acid] 10,124 (5.0%)	Ibuprofen 54,350 (4.2%)	Metoprolol succinate 16,843 (2.6%)	Diabetic oral feed 1 kcal/ml 5781 (3.4%)	Metronidazole 239,964 (4.7%)
**7**	Paracetamol with codeine 102,720 (4.5%)	Metoprolol succinate 94,759 (4.1%)	Diclofenac sodium 8832 (4.4%)	Clotrimazole 49,744 (3.9%)	Omeprazole 16,753 (2.6%)	Amino acid formula 5742 (3.4%)	Ferrous fumarate 222,571 (4.3%)
**8**	Diclofenac sodium 75,982 (3.4%)	Blood glucose diagnostic test strip 85,905 (3.8%)	Ciprofloxacin 8021 (4.0%)	Condoms 47,916 (3.7%)	Aspirin 15,885 (2.4%)	Paediatric oral feed with fibre 1.5 kcal/ml 5688 (3.3%)	Codeine phosphate 194,336 (3.8%)
**9**	Chlorhexidine gluconate 51,995 (2.3%)	Metformin hydrochloride 77,871 (3.4%)	Dexamethasone 6882 (3.4%)	Norethisterone 43,376 (3.4%)	Salbutamol 14,323 (2.2%)	Paediatric oral feed 1.5 kcal/ml 4988 (2.9%)	Levonorgestrel 157,870 (3.1%)
**10**	Tramadol hydrochloride 41,567 (1.8%)	Prednisone 76,888 (3.4%)	Sodium hyaluronate [Hyaluronic acid] 6035 (3.0%)	Ascorbic acid 40,912 (3.2%)	Metformin hydrochloride 13,843 (2.1%)	Renal oral feed 1.8 kcal/ml 4792 (2.8%)	Diclofenac sodium 157,023 (3.0%)

## Discussion

### Summary of findings

This study reported the first data describing all NMP in New Zealand providing an important baseline for future comparisons. The increasing contribution trend of nurse prescribers towards NZ NMP could be due to their new ‘registered nurse prescriber’ scopes of practice [23], a supportive regulatory authority, and streamlined education programmes, allowing increasing numbers of nurses prescribers (nurse practitioners and registered nurse prescribers) to prescribe in practice in NZ [24]. As New Zealanders make more use of some of the long-standing and new NMP providers such as nurse prescribers, the proportion of contributions towards NMP by some NMP providers such as dentists and midwives has decreased to some extent.

Only nurse prescribers and midwives prescribed for a higher proportion of Māori patients (21.6% and 17.6% respectively) than in the general New Zealand population (16.5%) [25]. However, overall it would appear that NMP providers were not providing healthcare to all communities with greater health needs, for example NMP providers prescribed for a lower proportion of patients of Pacific ethnicity than found in the general New Zealand population (8.1%) [25]. Midwives and nurse prescribers prescribed for a younger cohort of patients in NZ, with patient median ages (29 and 36 years respectively), slightly below the NZ national median age of 37.4 years [26].

Antibacterials and analgesics (including non-steroidal anti-inflammatory agents) were, on average, the most prescribed therapeutic groups by NMP providers. These two therapeutic groups were also in the top five groups prescribed by NZ GPs in the most recent statistics (2018) [27]. There is a lot of similarity in the top ten most prescribed medicines by NMP providers in this study and by GPs [27]. For example, amoxicillin and amoxicillin with clavulanic acid which ranked highly in NMP also featured in the top twenty most prescribed medicines by GPs [27]. The analgesics (including paracetamol, paracetamol with codeine, codeine, ibuprofen, diclofenac, and tramadol hydrochloride), featured in the top ten medicines prescribed by all NZ NMP providers overall, and all prescribers except dietitian prescribers. In contrast, paracetamol and ibuprofen were the only analgesics in the top twenty most prescribed medicines by GPs [27]. However, opioids may be lower on the list of most prescribed by GPs as they have a wider range of patients/conditions to treat. NZ data indicates that codeine and tramadol were the two most prescribed opioids between 2013 and 2017, with tramadol use increasing by 13% [28].

### Comparison with existing literature

It is difficult to compare the prescribing practice of the NZ NMP providers in this study as there a scarcity of published work about NMP providers’ practice in NZ and internationally. The number of original prescriptions written by non-medical prescribers in NZ is increasing and has surpassed the 1 million NMP prescriptions reported in earlier NZ research [8], with this study reporting 1,407,028 NMP prescriptions in 2016 and more than doubling to 3,101,161 NMP prescriptions by 2019.

Similar to NZ, UK research reported that nurse prescribers are the biggest contributor to NMP prescriptions in the UK, however UK nurse prescribers accounted for a much higher proportion (94.4%) of NMP prescriptions dispensed in primary care (93,102,682 out of 98,577,980 NMP prescriptions) for the 5 year period January 2011–December 2015 [29]. It is noted that in the UK, midwives are regulated by the Nursing and Midwifery Council UK and their prescribing is captured within nurse prescribing [30].

While pharmacist prescribers contributed modestly (0.25%) to all NZ prescribing, they were the fourth largest contributors to NMP prescriptions (9.0%) from 2016 to 2020 (Fig. 1), with a high of 12% in 2018. In contrast, pharmacist prescribers in the UK are the second largest contributor to NMP prescriptions in primary care producing 5.5% of all NMP prescriptions dispensed during January 2011–December 2015 [29]. While the UK percentage is smaller than that of pharmacist prescribers in NZ, the number of NMP prescriptions written by pharmacist prescribers is much higher in the UK (i.e. 5,454,942 out of 98,577,980 NMP prescriptions from 2011 to 2015) [29], than in NZ (i.e. 890,444 out of 10,274,324 NMP prescriptions from 2016 to 2020). Pharmacist prescribers in both countries have the potential to contribute more to NMP and overall prescribing in primary care.

There is only one existing NZ study about NMP practice with which this present study can be compared (Poot et al), however, that study only focused on nurse practitioner prescribing practice [10]. This present study’s nurse prescribers’ patient cohort is younger than seen in Poot et al’s study (median age = 40 years) [10]. One reason could be the broad practice scope of nurse practitioners compared to the primary care focus of the registered nurse prescribers. Poot et al found that nurse practitioners were prescribing for patients living in areas of more deprivation, to a higher proportion of Māori patients, and a lower proportion of Pacific people [10], which was similar to patient cohort findings for the nurse prescribers in the present study.

NZ research found three antibiotics (amoxicillin, amoxicillin with clavulanic acid, and flucloxacillin) in the top ten most prescribed medicines by nurse practitioners in 2013–2015 [10]. UK research also confirms that penicillins, which include amoxicillin and amoxicillin with clavulanic acid, are the most prescribed antibacterial class for all NMPs overall, and nurse and pharmacist prescribers [29]. Given the increasing issue of antimicrobial resistance, further examination around the appropriateness of this prescribing is warranted as this is largely unknown. Earlier NZ research evaluating antibiotic prescribing found that generally NZ dentists followed clinical guidelines [12], however recent Australian research concluded the overuse of antibiotics by Australian dentists for both therapeutic and non-therapeutic reasons [31]. This Australian research also found that a small proportion of general dentists prescribed diclofenac, codeine, and tramadol, which were considered inappropriate analgesics according to their Australian oral and dental therapeutic guidelines [31]. While there are no specific dentist guidelines in NZ for prescribing analgesics, this present NMP study found that NZ dentists were prescribing diclofenac, codeine, and tramadol which were considered inappropriate analgesics according to Australian guidelines.

### Implications for policy and practice in NZ

The larger urban DHBs account for 48.3% of NZ NMP patients as they have bigger population bases. Based on current prescribing practice of the overall NMP service, there is a great opportunity for NZ NMP providers, including pharmacist prescribers, to contribute to communities with greater health needs, including Māori and Pacific communities [32, 33]. Rural NZ struggles with adequate workforce and inequity of access to healthcare [34], and rural DHBs often have higher proportions of patients who experience an unmet need for primary healthcare (e.g. a higher proportion of Māori and Pacific patients) [35]. While it is acknowledged that the rural DHBs account for smaller proportions of all NMP patients in this study due to their smaller population bases, NMP services could be further utilised to reach communities who are experiencing unmet primary healthcare needs in rural NZ.

The high prescribing of analgesics and antibiotics by non-medical prescribers suggest that they are dealing with a high number of acute conditions. These examples could signal that non-medical prescribers are prescribing within their scope such as dentists treating oral/dental infections and conditions that required pain relief. It also suggests that non-medical prescribers could be taking some of the burden from GPs and be helping to improve medicines access equity for acute conditions. UK research has indicated that NMP has made better use of health professionals’ skills (including nurse and pharmacist independent prescribers) to reduce workload pressures of GPs [36–38].

NZ pharmacist prescribers prescribed more preventative agents (nicotine replacement therapy), and immunisations (influenza vaccine). UK pharmacists are also increasingly prescribing the influenza vaccine [39] and in 2018, pharmacists in Canada also delivered 34% of adult influenza doses [40]. The current uptake of NZ pharmacist prescribers for preventative agents demonstrates that they could be further utilised to prescribe preventative agents, including a wider range of funded immunisations in NZ. Older people (65 years and over) make up 15% of the NZ population, use 42% of health services, and over the last 10 years spending on services for older people is increasing faster than other expenses [41] Current NZ pharmacist prescriber practice indicates that they prescribe more medicines for long term conditions and for a higher proportion of older patients. NZ pharmacist prescribing services could be improved in primary care to help manage the increasing prescribing burden associated with long term conditions and an aging population. Canadian research has demonstrated the value of pharmacist prescribing services to manage long term conditions such as hypertension and cardiovascular risk [42, 43], which increase in prevalence as people age.

Understanding NMP practice in NZ identifies areas that require sufficient initial and continuing education. NZ is a relatively high user of antibiotics [44, 45], and overuse of antibiotics is a key factor [46, 47] in the global health problem of antibiotic resistance [48, 49]. Identifying that antibiotics are commonly prescribed by NMPs suggests that further research is required to evaluate the appropriateness of this prescribing. It also highlights the need for adequate education about antimicrobial stewardship to ensure that NMPs are not contributing to antibiotic resistance [50]. Appropriate analgesic prescribing is also important, especially with the concerns internationally about rising prescription opioid use and misuse [51]. Prescribers need to be fully aware of opioid-related risks and adverse effects, as well as the potential for opioid misuse [52–54] while optimising pain relief for patients.

### Strengths and limitations

A strength of this study is the large data set available for analysis, with dispensing, prescriber, patient, and medicines details. The data set contained comprehensive sociodemographic details for each patient and captures > 96% of the New Zealand population. It is acknowledged that dispensing may not fully reflect prescribing, as patients who had a prescription issued but did not take it to a pharmacy for dispensing, are not captured in this study. The Pharmaceutical Collection data did not differentiate between pharmacists prescribing funded medicines as registered pharmacist prescribers and via the pharmacist only medicines classification. This study captured both nurse practitioner and registered nurse prescribing within the ‘nurse prescribing’ umbrella term.

## Conclusions

All the NMP professions in NZ appear to be prescribing medicines within their usual practice. While there is modest growth in this service, NZ NMP still makes very small contributions to overall prescribing in primary care. Nurse prescribers are the largest contributor to NMP, and other NMP providers including pharmacist prescribers have the potential to contribute more to this service.

There is some indication that NMP providers could be alleviating the prescribing burden on GPs and improving access by prescribing for acute conditions that require pain relief or antibiotics. However, overall all NMP providers could be better utilised to provide prescribing services to our communities with greater health needs and improve access to these healthcare services. NMP providers have the potential to improve healthcare service delivery to cope with unmet health needs in rural NZ, a sector that struggles to meet health needs due to workforce constraints and inequity of access. The variation of NMP service provision across the different DHBs in NZ indicates that the NMP service has been well integrated in certain geographic areas, and this variation could be due to better support and funding in some DHBs compared to others. This study highlights the importance of identifying what medicines NMP providers are prescribing, to ensure adequate education and continuing professional development to support best practice and appropriate prescribing decisions. Further research is needed to determine the impact of NMP on patient outcomes and access to healthcare.

As NZ seeks to cope with the increasing demand for health services, factors such as workforce constraints have the potential to affect the availability of the usual GP prescriber in primary care. NZ has to make better use of the skills of other health professionals, and must consider new models of care and service delivery to meet these increasing healthcare needs. This study highlights that NMP has been implemented in NZ, but it has not yet become mainstream healthcare practice. This work provides a baseline to evaluate the NMP service moving forward and contribute to policy development. Improved implementation and integration of primary care NMP services can enable continued access and reduce inequity to prescribing services and medicines for our communities.

## Data Availability

The data sets generated and analysed during this study are not publicly available due to constraints imposed in the Ethics process, but are available from the corresponding author on reasonable request subject to approval.

## Abbreviations

DHB: District Health Board
GP: General practitioner
GPs: General practitioners
IQR: Inter-quartile range
MELAA: Middle Eastern, Latin American, and African
NMP: Non-medical prescribing
NHI: National Health Index
NZ: New Zealand
NZDep2013: New Zealand Deprivation Index 2013
PHO: Primary Health Organisation
SD: Standard deviation
UK: United Kingdom
